# Potato Protein-Based Vegan Burgers: Discovering the Health-Promoting Benefits and Impact on the Intestinal Microbiome

**DOI:** 10.3390/nu18010160

**Published:** 2026-01-03

**Authors:** Przemysław Łukasz Kowalczewski, Małgorzata Gumienna, Paweł Jeżowski, Michał Świątek, Barbara Górna-Szweda, Iga Rybicka, Millena Ruszkowska, Maciej Ireneusz Kluz, Matteo Bordiga

**Affiliations:** 1 Collegium Medicum, Andrzej Frycz Modrzewski Krakow University, 30-705 Kraków, Poland; mkluz@uafm.edu.pl; 2 Department of Food Technology of Plant Origin, Poznań University of Life Sciences, 60-624 Poznań, Poland; malgorzata.gumienna@up.poznan.pl (M.G.); barbara.gorna@up.poznan.pl (B.G.-S.); 3 Institute of Chemistry and Technical Electrochemistry, Poznan University of Technology, 60-965 Poznań, Poland; pawel.jezowski@put.poznan.pl; 4 Ekosystem-Nature’s Heritage Association, Institute of Microbial Technologies, 62-700 Turek, Poland; michal.swiatek@itm.turek.pl; 5 Department of Technology and Instrumental Analysis, Poznań University of Economics and Business, 61-875 Poznań, Poland; iga.rybicka@ue.poznan.pl; 6 Department of Quality Management, Gdynia Maritime University, 81-225 Gdynia, Poland; m.ruszkowska@wznj.umg.edu.pl; 7 School of Medical and Health Sciences, Vizja University, 01-043 Warszawa, Poland; 8 Department of Pharmaceutical Sciences, Università degli Studi del Piemonte Orientale “A. Avogadro”, Largo Donegani 2, 28100 Novara, Italy; matteo.bordiga@uniupo.it

**Keywords:** potato protein, plant-based burger, intestinal microbiota, cytotoxicity, *β*-glucuronidase, vegan meat analogues, gut health

## Abstract

Introduction: The increasing global interest in plant-based diets has led to the development of innovative meat analogs that not only mimic the sensory properties of traditional products but may also offer potential health benefits. In this study, we investigated the nutritional characteristics and biological activity of potato protein-based vegan burgers (PBBs) enriched with plant-derived iron and fiber sources. Methods: The burgers were subjected to *in vitro* gastrointestinal digestion, followed by evaluation of their cytotoxic potential against human intestinal cancer cell lines (Caco-2 and HT-29) and normal colon epithelial cells (CCD 841 CoN). Additionally, their influence on the intestinal microbiota composition and enzymatic activity of *β*-glucosidase and *β*-glucuronidase was assessed. Results: PBBs demonstrated favorable nutritional profiles, high protein and fiber contents, and a balanced fatty acid ratio (*n*-6/*n*-3). After digestion, bioaccessible fractions showed selective cytotoxicity toward cancer cells, while maintaining safety for normal intestinal cells. Furthermore, PBBs modulated the gut microbiota by promoting the growth of beneficial genera (*Lactobacillus*, *Bifidobacterium*) and reducing potentially harmful Enterobacteriaceae, accompanied by decreased *β*-glucuronidase activity. Conclusions: These findings suggest that potato protein-based burgers could represent a functional plant-based alternative to conventional meat products, contributing to intestinal health and potentially reducing colorectal cancer risk.

## 1. Introduction

In recent years, the global market for plant-based products has experienced remarkable growth, driven by consumer awareness of health, environmental sustainability, and ethical concerns, coupled with rapid technological progress in food innovation. Market analyses show that though plant-based foods currently represent less than 4% of the total global protein consumption, the sector has grown at a compound annual rate two to three times higher than that of more traditional meat and poultry industries [1,2]. The strongest dynamics can be seen in beverages, dairy alternatives, and plant-based meat analogs (PBMAs). Among these, PBMAs have recently received particular attention from both consumers and the scientific community because their potential to replace animal-derived products while maintaining similar sensory and nutritional attributes is enormous [3,4,5]. This growth depicts developing confidence in the economic and functional potential of this market, reflected in surging investments, new product launches, and biotechnology-driven start-ups [5,6]. Although PBMAs remain a niche segment and their price is often sensitive compared to animal meat, further development offers promising opportunities for nutritional profile improvement, decrease in environmental impact, and supporting the shift toward more sustainable dietary patterns [7,8,9].

There is strong evidence from multiple mechanistic and epidemiological studies that points to the regular consumption of meat—particularly processed and high-temperature cooked red meat—as increasing the risk of colorectal cancer. Meat may promote intestinal carcinogenesis through overlapping chemical, oxidative, inflammatory, and microbiome-mediated pathways leading to DNA damage and epithelial proliferation [10,11,12]. Heme iron, abundant in red meat, catalyzes endogenous formation of *N*-nitroso compounds (NOCs) and enhances lipid peroxidation in the gut lumen, generating genotoxic and cytotoxic species [10,11]. Additional carcinogenic factors include preformed NOCs derived from curing and preservatives, as well as cooking-induced mutagens such as heterocyclic aromatic amines (HCAs) and polycyclic aromatic hydrocarbons (PAHs), which form bulky DNA adducts (e.g., O^6^-carboxymethylguanine) that initiate mutational events [12,13,14]. High intake of animal fat further promotes the bacterial conversion of bile acids to secondary genotoxic forms, while meat-derived compounds alter the gut microbiota by favoring pro-tumorigenic species such as *Fusobacterium* and certain *E. coli* strains [15,16]. Moreover, large cohort studies confirm that high processed-meat intake increases colorectal cancer risk by approximately 20–50%, and higher meat consumption correlates with molecular markers of endogenous *N*-nitrosation and oxidative stress [12,14,17]. Collectively, these findings justify growing scientific and public interest in plant-based meat alternatives as potential dietary strategies to reduce exposure to heme-driven nitrosation, cooking-derived mutagens, and microbiome dysbiosis associated with traditional meat consumption [15,17,18].

Potato protein recovered from industrial potato fruit juice represents a particularly attractive ingredient for vegan food production, as it combines technological robustness with high nutritional and health-promoting potential [19,20,21,22]. Potato processing streams provide a protein fraction with an essential amino acid profile and DIAAS values that are comparable to, or in some cases approaching, reference proteins, including soy, making it highly suitable as a primary protein source in plant-based formulations [23,24]. In addition to comparisons with commonly used plant proteins, potato protein has increasingly been evaluated against animal-derived proteins, including egg white and dairy proteins. Recent studies indicate that potato protein exhibits a highly favorable amino acid profile, high digestibility, and techno-functional properties—particularly emulsifying and foaming capacities—that are comparable to those of selected animal proteins [25]. These characteristics position potato protein not only as an alternative to conventional plant protein ingredients but also as a sustainable substitute for animal-derived proteins in food formulations. Such comparisons further highlight the relevance of potato protein in the development of next-generation plant-based foods with improved nutritional and functional performance. Moreover, potato proteins are rich in branched-chain amino acids, which are important for muscle protein synthesis and metabolic health, thereby supporting the development of vegan products targeted at physically active and clinically vulnerable consumers [23]. Beyond basic nutrition, controlled extraction and enzymatic or membrane-assisted processing of potato proteins yield peptide fractions with documented antioxidant activity and bioactive properties, including selective anti-cancer effects and potential cardioprotective actions, positioning potato-derived ingredients as candidates for functional and nutraceutical foods [26,27]. From a sustainability perspective, upcycling waste potato juice into high-value protein aligns with circular economy principles and markedly improves the environmental performance of the starch industry by transforming an effluent liability into a nutrient-dense, health-oriented ingredient for meat analogs, dairy alternatives and other plant-based foods [23,28,29,30].

Therefore, in the present study, we aimed to comprehensively characterize potato protein-based vegan burgers (PBBs) as a potentially health-promoting alternative to meat products, with a specific focus on intestinal endpoints. Building on previously described formulation and nutritional characteristics of these burgers, we subjected them to standardized *in vitro* gastrointestinal digestion and (i) evaluated the cytotoxic and cytostatic effects of the resulting digests toward human colorectal cancer cell lines (Caco-2, HT-29) in comparison with normal colon epithelial cells (CCD 841 CoN), (ii) assessed their impact on selected gut microbiota groups and on the activity of microbial enzymes implicated in colorectal carcinogenesis (*β*-glucuronidase and *β*-glucosidase), and (iii) verified microbiological safety and stability of the products under refrigerated storage.

## 2. Materials and Methods

### 2.1. PBBs Manufacturing

PBBs were prepared in accordance with the procedure described in detail previously [31]. The detailed composition of the analyzed burgers is presented in Table S1. Briefly, a blend of potato (70%) (Poznań University of Life Sciences, Poznań, Poland), rice (20%) (Beneo, Veendam, Belgium), wheat (5%) (Viresol, Visonta, Hungary), and pea protein (5%) (Brenntag, Kędzierzyn-Koźle, Poland) was mixed with water (1:6 *w*/*v*), homogenized, and baked in metal molds at 120 °C for 2 h, then cooled and stored at 4 °C. An oil blend of 55% rice bran (Kasisuri, Bangkok, Thailand) and 45% rapeseed (ZT Kruszwica, Kruszwica, Poland) oils was prepared to achieve a 5:1 *n*-6 to *n*-3 fatty acid ratio. The coconut oil (Bazar Zdrowia, Skawa, Poland) was added for structure. The protein base was mixed with oils, followed by dry ingredients, water, and vinegar, and then vacuum-packed and stored for 24 h at 4 °C. Afterward, 75 g burgers were formed and thermally processed at 160 °C for 30 min. The PBBs were vacuum-sealed and stored at 4–6 °C until analysis.

### 2.2. Cytotoxic Activity

An *in vitro* digestion procedure without addition of intestinal microflora was performed as follows: 10 g of the freeze-dried, ground sample was placed into glass lab containers, and distilled water was added to reach a final volume of 100 mL. The pH was brought to 2.0 by adding 4 M hydrochloric acid (Sigma-Aldrich, Poznań, Poland). To simulate gastric conditions, pepsin (1.92 mg/g) (Sigma-Aldrich) dissolved in 0.1 M hydrochloric acid was introduced, followed by a 2 h incubation period. Afterward, the pH was raised to between 7.2 and 7.4 using 1 M sodium bicarbonate (Sigma-Aldrich) to replicate small intestinal conditions. A solution containing pancreatic extract (0.4 mg/g) (Sigma-Aldrich) and bile salts (2.4 mg/g) (Sigma-Aldrich), dissolved in 0.1 M sodium bicarbonate, was then added to mimic small intestine digestion. The mixture was incubated for 2.5 h at 37 °C in a shaking water bath. Finally, the samples were frozen at −80 °C and subsequently freeze-dried. This simplified gastrointestinal digestion model, excluding the colonic fermentation stage and intestinal microbiota, was intentionally applied to obtain well-defined and reproducible post-digestion fractions suitable for cell culture experiments. The absence of microbial metabolites and bacterial components minimizes potential interference with cell viability, proliferation, and metabolic assays, which is particularly important when using epithelial cell lines. The cytotoxic potential of freeze-dried, digested PBBs was determined in cancer Caco-2 (ATCC^®^ HTB-37™), HT-29 (ATCC^®^ HTB-38™) and normal CCD 841 CoN (ATCC^®^ CRL-179™) human cell lines, purchased from American Type Culture Collection (ATCC, Manassas, VA, USA). The method described earlier was used [32]. In cytotoxicity testing, cells were initially seeded in 96-well plates at a density of 1.5 × 10^4^ cells/cm^2^. Following 24 h incubation, cultures were treated with tested samples for 48 h. PBBs were tested across a range enabling dose–response modeling. Treatment duration of 48 h was optimized based on cell proliferation rates, ensuring consistent conditions across normal and cancer cell lines. Cell viability and metabolic activity were assessed using the MTT (3-(4,5-dimethylthiazol-2-yl)-2,5-diphenyltetrazolium bromide) (Sigma-Aldrich) assay for its sensitivity and repeatability. Absorbance was measured at 570 nm and 690 nm after incubation with MTT solution (5 mg/mL) and extraction of formazan crystals. Finally, calculation of cytotoxic doses for each compound were performed.

### 2.3. Determination of β-Glucuronidase (EC 3.2.1.31) and β-Glucosidase (EC 3.2.1.21) Activities

The *in vitro* digestion of PBBs before analysis was carried out in accordance with the methodology described in Kowalczewski et al. [33], employing a comprehensive gastrointestinal digestion model that included the colonic fermentation stage with intestinal microbiota. The inclusion of gut microflora was essential, as both *β*-glucuronidase and *β*-glucosidase are predominantly of microbial origin and their activity reflects microbiota-driven metabolic processes rather than host-mediated digestion alone. The determination was conducted based on the modified methods of Djouzi and Andrieux [34] and Kapnoor and Mulimani [35]. The sample was prepared by collecting 200 μL from the bioreactor, to which 1500 μL of phosphate buffer (pH 7.0) and NaCl (0.1 M) were added. The samples were shaken for 1 h, then centrifuged for 15 min at 4000× *g* to obtain the supernatant. To 200 μL of the supernatant, 200 μL of *p*-nitrophenyl-*β*-D-glucuronide (Sigma-Aldrich) (for the determination of *β*-glucuronidase) or *p*-nitrophenyl-*β*-D-glucopyranoside (Sigma-Aldrich) (for the determination of *β*-glucosidase) were added, followed by incubation for 2.5 h at 40 °C. The reaction was stopped by adding 2000 μL of sodium carbonate (Sigma-Aldrich). Absorbance was measured at 420 nm. Enzymatic activity was expressed as μmol of product (*p*-nitrophenol) formed per minute per gram of digested content (U/g digested sample).

### 2.4. Impact of PBBs on Gut Microbiota

To determine the impact of PBBs on microbial growth, control inoculations were performed 2 h after introducing the microorganisms (at pH 7.4, simulating the small intestine) and at the end of the digestion process (after 21 h). The human intestinal microflora was introduced into the experimental setup [33]. The targeted microorganism groups were: *Enterobacteriaceae* (using MacConkey selective medium), *Lactobacillus* (using MRS agar medium), *Enterococcus* (using agar with kanamycin, esculin, and sodium azide), and *Bifidobacterium* (using Garche medium). All media were purchased from Becton Dickinson (Franklin Lakes, NJ, USA). The inoculated media were incubated anaerobically at 37 °C for 48 to 72 h, depending on the microorganism group. The number of viable bacterial cells was determined using Koch’s plate method.

### 2.5. Analysis of Microbiological Quality During Storage

Microbiological stability and safety of the products were assessed during storage at 3–8 °C in the absence of light and air for 22 days. Analyses were carried out in accordance with ISO standards.

The microbiological evaluation aimed to verify product safety (*Salmonella* spp., *Listeria monocytogenes*, *Clostridium perfringens*), process hygiene (*Enterobacteriaceae*, staphylococci), and storage stability (total viable count, yeasts, and molds).

The presence of *Salmonella* spp. and *Listeria monocytogenes* was determined according to PN-EN ISO 6579-1:2017 [36] and PN-EN ISO 11290-1:2017 [37], respectively. Representative 25 g samples were homogenized in buffered peptone water (Oxoid), subjected to selective enrichment, and plated on differential agars (XLD, Chromagar Salmonella Plus, ALOA, and Oxford). Typical colonies were confirmed by biochemical and serological identification, including sugar fermentation profiles, hemolysis, and Gram staining.

Quantitative analyses were performed by homogenizing 10 g of each sample in 90 mL of peptone water and preparing ten-fold serial dilutions. Enumeration of total viable microorganisms (PN-EN ISO 4833-1:2013) [38], *Enterobacteriaceae* (PN-EN ISO 21528-2:2017) [39], *Clostridium perfringens* (PN-EN ISO 15213-2:2024) [40], coagulase-positive staphylococci (PN-EN ISO 6888-1:2022) [41], and yeasts and molds (PN-EN ISO 21527-1:2009) [42] was performed using standard selective and differential agars (PCA, VRBG, TSC, BP, and DRBC, respectively). All media were purchased from Becton Dickinson (Franklin Lakes, NJ, USA). Incubation was conducted under aerobic or anaerobic conditions as appropriate.

### 2.6. Statistical Analyses

The experimental data, expressed as mean ± SD, underwent one-way analysis of variance (ANOVA), followed by Tukey’s post hoc test to identify statistically homogeneous groups at α = 0.05 significance level using Statistica 13.3 (TIBCO Software Inc., Palo Alto, CA, USA).

## 3. Results

### 3.1. Basic Nutritional Composition

The nutritional value of the produced burgers was previously described and discussed in a published article [31]. However, the results are presented below, which are essential for explaining the observed changes (Table 1).

### 3.2. Cytotoxicity of PBBs

The digested fractions of all potato protein-based burgers (PBBs) exhibited dose-dependent cytotoxic activity toward colorectal cancer cell lines (HT-29 and Caco-2), while maintaining substantially lower cytotoxicity toward normal colon epithelial cells (CCD 841 CoN). The IC_50_ values for HT-29 cells ranged from 3.11 to 4.17 mg/mL (Table 2), while for Caco-2 cells the values were slightly lower, between 2.98 and 3.90 mg/mL, indicating higher sensitivity of Caco-2 cells to the digested material. In contrast, non-cancer colon epithelial cells (CCD 841 CoN) showed markedly higher IC_50_ values, demonstrating a protective cytoselectivity window between cancer and non-cancerous cells. PBB4 showed the strongest anti-cancer effect with the lowest IC_50_ values toward both HT-29 (3.11 mg/mL) and Caco-2 (2.98 mg/mL) cells. This variant contains iron(II) sulfate instead of ferritin and employs oat fiber, which may modulate the release of bioactive compounds during digestion. Contrarywise, PBB1 and PBB3—formulated with potato fiber—showed slightly weaker but still distinct cytotoxic potential.

### 3.3. Modulation of Intestinal Microflora and Microbial Enzyme Activities

The simulated gastrointestinal digestion of PBBs resulted in pronounced yet formulation-dependent modulation of both microbial composition and bacterial enzymatic activity during the colonic phase. Across all variants, fermentation promoted substantial proliferation of *Lactobacillus* and *Bifidobacterium*, which reached levels approaching 9–11 log CFU/mL (Table 3). This consistent increase indicates that the PBBs provided substrates readily utilized by saccharolytic bacterial taxa. The effect is likely attributable to the combined presence of fermentable potato-derived fibers, residual starch fractions, and phenolic compounds released during digestion. The development of *E. coli* and *Enterococcus* showed greater variability among formulations. In PBB1 and PBB3, both taxa declined or remained stable during the colonic phase. In contrast, PBB2 and PBB4 (enriched with oat fiber) supported moderate growth of these genera, yet without disrupting the overall dominance of *Lactobacillus* and *Bifidobacterium*.

Changes in microbial composition were closely mirrored by shifts in bacterial enzymatic activity. Both *β*-glucuronidase and *β*-glucosidase exhibited the highest values immediately after inoculation, decreased during the small-intestinal phase, and reached their minimum at the onset of colonic fermentation (Table 4). During the 18 h fermentation period, *β*-glucosidase activity increased substantially in PBB3 and PBB4, whereas *β*-glucuronidase increased more moderately. In contrast, *β*-glucuronidase activity remained at levels considerably lower than those associated with microbiota states linked to carcinogen reactivation or intestinal dysbiosis.

### 3.4. Microbiological Safety

Microbiological quality assessment conducted over a 22-day refrigerated storage period (3.0–8.0 °C, protected from light and air) demonstrated that all evaluated product variants maintained excellent microbial stability and safety throughout the entire testing interval. Across all time points, no statistically or technologically relevant increases were detected in any of the monitored microbiological parameters. Total aerobic mesophilic counts remained consistently low, with values ranging from 2.2 to 2.9 log CFU/g, indicating minimal microbial proliferation during storage. Counts of *Enterobacteriaceae*, yeasts and molds, coagulase-positive *Staphylococcus* spp., and *Listeria monocytogenes* were uniformly below the quantification threshold of 1.0 log CFU/g, suggesting effective inhibition of microorganisms commonly associated with hygiene lapses or early spoilage (Table 5). 

Furthermore, *L. monocytogenes* and *Salmonella* spp. were not detected in any of sample at any measurement point. The absence of these pathogens, combined with consistently low indicator organism counts, attests to both the microbiological integrity of the formulations and the effectiveness of the applied production and packaging conditions. The results clearly demonstrate that the investigated PBBs exhibit robust microbiological safety and remain stable throughout 22 days of refrigerated storage, supporting their suitability for commercial distribution and consumption within this timeframe.

## 4. Discussion

The present study demonstrated that PBBs exhibit favorable nutritional characteristics and biologically relevant activity following *in vitro* gastrointestinal digestion. A key finding was the selective cytotoxicity of digested PBB fractions toward colorectal cancer cell lines (Caco-2 and HT-29), accompanied by lower toxicity toward normal colon epithelial cells. This observation is consistent with earlier reports describing antiproliferative and pro-apoptotic effects of potato-derived peptides and phenolic compounds obtained from potato juice and processing by-products [43,44]. Importantly, the present data extend these findings to a complex, ready-to-eat plant-based meat analog, rather than isolated extracts or fractions.

To better contextualize these results, it is important to compare PBBs with other plant-based meat analogs (PBMAs), which are predominantly formulated using soy, pea protein, wheat gluten, mycoprotein, or mixed legume–cereal matrices [2,4,5]. Although many PBMAs successfully mimic the sensory properties of meat, their nutritional and biological effects vary substantially depending on protein source, fiber composition, degree of processing, and fortification strategy [7,8,9]. Highly refined PBMAs based mainly on protein isolates often contain limited fermentable fiber and polyphenols, which may restrict their capacity to beneficially modulate gut microbiota or generate bioactive metabolites during digestion.

Soy- and pea-based burgers are the most extensively studied PBMAs. While these products can provide adequate protein quality and support satiety, their effects on gut microbiota are inconsistent and frequently depend on additional fiber enrichment [2,5]. Several studies have shown that diets rich in isolated plant proteins, similarly to animal protein-rich diets, may promote proteolytic fermentation and increase microbial enzyme activities associated with carcinogen activation when fermentable substrates are limited [14,45,46,47]. In contrast, PBMAs enriched with intact fibers, resistant starch, or polyphenol-rich ingredients have been shown to stimulate saccharolytic bacteria such as *Lactobacillus* and *Bifidobacterium*, contributing to improved colonic metabolic profiles [48,49,50].

In this context, the PBBs investigated in the present study align more closely with fiber-enriched and minimally processed PBMAs. All formulations consistently promoted the growth of *Lactobacillus* and *Bifidobacterium* during simulated colonic fermentation, reaching levels comparable to those reported for diets rich in whole plant foods and resistant starch [32,48,49]. The observed microbiota modulation contrasts with patterns typically associated with red and processed meat consumption, which has been linked to increased abundance of *Enterobacteriaceae*, *Fusobacterium*, and elevated *β*-glucuronidase activity [12,14,15,16,17].

The maintenance of *β*-glucuronidase activity at relatively low physiological levels across all PBB variants is particularly relevant from a colorectal cancer prevention perspective. Excessive *β*-glucuronidase activity is known to promote the reactivation of detoxified carcinogens and has been repeatedly associated with meat-rich and high-fat diets [14,45,46,47,51]. The present results indicate that the potato protein–fiber matrix does not favor such detrimental microbial metabolism, even in formulations containing added iron. This finding supports epidemiological and mechanistic evidence suggesting that plant-based alternatives lacking heme iron may help avoid pro-carcinogenic pathways linked to conventional meat intake [10,11,12,18]. *β*-glucosidase activity increased during colonic fermentation, particularly in PBB3 and PBB4. Unlike *β*-glucuronidase, *β*-glucosidase is generally considered beneficial, as it mediates the release of phenolic aglycones from dietary glycosides, thereby enhancing the bioavailability and biological activity of polyphenols [50,52,53]. Similar effects have been described for cereal brans, legume hulls, and polyphenol-rich plant matrices, where increased *β*-glucosidase activity correlates with enhanced antioxidant and antiproliferative effects of digested material [52,53,54]. The present data suggest that PBBs support a microbiota-driven activation of bioactive compounds, which may partly explain the selective cytotoxicity observed toward colorectal cancer cell lines.

The antiproliferative effects of digested PBBs compare favorably with those reported for other plant-based matrices evaluated *in vitro*. Previous studies on legume-based foods, cereal fractions, and mushroom-derived meat analogs have reported IC_50_ values in a similar or higher concentration range, often without clear selectivity toward cancer cells [53,54]. Notably, PBB4 exhibited the lowest IC_50_ values against both HT-29 and Caco-2 cells. This formulation contained oat fiber and ionic iron rather than ferritin-bound iron, which may have influenced peptide release, oxidative signaling, and microbial metabolism during digestion and fermentation. Oat-derived *β*-glucans and mixed-linkage polysaccharides have been shown to enhance fermentability and modulate redox-sensitive pathways relevant to cancer cell proliferation [48,49,55,56].

Compared with soy and wheat gluten, potato protein offers several additional advantages relevant to PBMA development. Potato protein is characterized by high digestibility, a balanced essential amino acid profile, and relatively low allergenicity [19,20,21,22,23]. Moreover, the use of potato protein recovered from industrial potato juice aligns with circular economy principles and improves the sustainability profile of PBMAs by upcycling an underutilized by-product [28,29,30]. In contrast to some commercially available PBMAs that rely heavily on emulsifiers, stabilizers, and flavoring systems that may negatively affect gut barrier function, the PBB formulations were based on relatively simple ingredient compositions dominated by protein, fiber, and vegetable oils with a favorable *n*-6/*n*-3 fatty acid ratio.

The excellent microbiological stability of PBBs during refrigerated storage supports their technological feasibility as ready-to-eat products. Similar stability has been reported for other fiber-rich plant-based foods but remains a challenge for highly hydrated PBMAs lacking sufficient thermal or packaging control [28,31,57]. Together, these results indicate that PBBs combine nutritional adequacy, microbiological safety, gut microbiota modulation, and selective anticancer activity. Overall, when positioned within the broader spectrum of plant-based meat analogs, potato protein-based burgers emerge as a distinctive category that not only substitutes meat from a sensory and nutritional perspective but may also confer additional gut health–related benefits. The absence of heme iron, presence of fermentable fibers, and generation of bioactive digestion products differentiate PBBs from both conventional meat products and many existing PBMAs, supporting their potential role in dietary strategies aimed at reducing colorectal cancer risk.

## 5. Limitations and Future Perspectives

The present study has several limitations that should be acknowledged. All biological effects were assessed using *in vitro* models, including simulated gastrointestinal digestion, colorectal cancer cell lines, and fecal microbiota fermentation systems. While these approaches provide valuable mechanistic insight, they cannot fully capture the complexity of human digestion, host–microbiota interactions, or systemic responses. Therefore, the observed cytotoxic selectivity and microbiota modulation should be considered preliminary. Additionally, the microbiota experiments relied on a simplified fermentation model and fecal material from a limited donor source, which does not reflect inter-individual variability. Moreover, cancer cell lines do not reproduce the heterogeneity and microenvironment of colorectal tumors *in vivo*.

Future research should focus on *in vivo* validation, including animal models and human dietary intervention studies, to confirm the health relevance, bioavailability, and safety of potato protein-based burgers. Further studies exploring underlying molecular mechanisms, long-term consumption effects, and consumer-relevant product optimization will be essential to support their positioning as functional plant-based foods.

## 6. Conclusions

The present study demonstrates that PBBs are microbiologically stable products with favorable nutritional and functional characteristics and meaningful biological activity. All formulations remained free of pathogenic microorganisms throughout refrigerated storage, confirming their safety and technological robustness. Following simulated gastrointestinal digestion, PBBs generated bioaccessible fractions that selectively reduced the viability of colorectal cancer cells while preserving normal epithelial cells, reflecting the synergistic action of potato proteins, dietary fibers, and phenolic constituents. Additionally, all variants modulated the intestinal microbiota in a beneficial manner by stimulating saccharolytic taxa such as *Lactobacillus* and *Bifidobacterium*, maintaining *β*-glucuronidase activity at safe physiological levels, and supporting *β*-glucosidase activity linked to the release of bioactive phenolic aglycones. However, the biological effects were observed under *in vitro* conditions and should therefore be regarded as preliminary. These combined effects indicate that PBBs may contribute to gut microbial homeostasis and potentially reduce colorectal cancer risk through diet-associated mechanisms. Overall, the study supports the positioning of PBBs as functional plant-based foods with promising health-related properties, meriting further *in vivo* validation and long-term dietary assessments.

## 7. Patents

The results presented in this article were used to prepare a patent application to The Patent Office of the Republic of Poland (patent application No. P.446897, dated 29 November 2023).

## Figures and Tables

**Table 1:** Nutrients and energy values of PBBs.

Parameters	Sample			
	PBB1	PBB2	PBB3	PBB4
Protein content (g/100 g)	21.59 ± 0.35 ^a^	21.66 ± 0.86 ^a^	22.16 ± 0.95 ^a^	20.80 ± 1.06 ^a^
Fat content (g/100 g)	9.63 ± 0.77 ^a^	9.65 ± 0.60 ^a^	9.62 ± 0.63 ^a^	10.03 ± 0.92 ^a^
Fiber content (g/100 g)	8.35 ± 0.18 ^b^	9.20 ± 0.03 ^a^	8.42 ± 0.37 ^b^	9.08 ± 0.11 ^ab^
IDF (g/100 g)	7.45 ± 0.20 ^b^	8.08 ± 0.03 ^a^	7.54 ± 0.39 ^b^	8.05 ± 0.13 ^a^
SDF (g/100 g)	0.90 ± 0.03 ^b^	1.12 ± 0.02 ^a^	0.88 ± 0.03 ^b^	1.03 ± 0.05 ^ab^
Carbohydrate content (g/100 g)	10.13 ± 0.09 ^b^	7.65 ± 1.52 ^c^	12.34 ± 0.37 ^a^	11.22 ± 1.31 ^ab^
Mineral content (g/100 g)	7.10 ± 0.08 ^b^	6.96 ± 0.10 ^b^	7.56 ± 0.07 ^a^	6.50 ± 0.06 ^c^
Energy value (kcal/100 g)	248.0 ± 7.5	237.9 ± 2.9	248.9 ± 4.4	245.0 ± 7.2

Values marked with the same lowercase letter in the row do not differ significantly *p* > 0.05. Source: Ref. [31], based on the CC-BY license. IDF—insoluble dietary fiber; SDF—soluble dietary fiber.

**Table 2:** Cytotoxic activity against selected cell lines (mg/mL).

Cell Lines	Samples	IC_10_	IC_50_	IC_90_
HT-29	PBB1	1.801 ± 0.144 ^ab^	3.892 ± 0.211 ^a^	9.381 ± 0.240 ^a^
	PBB2	2.004 ± 0.174 ^a^	4.170 ± 0.204 ^a^	9.804 ± 0.695 ^a^
	PBB3	1.911 ± 0.175 ^a^	3.992 ± 0.274 ^a^	9.692 ± 1.030 ^a^
	PBB4	1.624 ± 0.105 ^b^	3.112 ± 0.142 ^b^	8.211 ± 0.552 ^b^
Caco-2	PBB1	1.642 ± 0.175 ^b^	3.076 ± 0.164 ^b^	7.192 ± 0.209 ^b^
	PBB2	1.913 ± 0.221 ^a^	3.901 ± 0.203 ^a^	7.906 ± 0.374 ^a^
	PBB3	1.739 ± 0.213 ^b^	3.556 ± 0.192 ^ab^	7.894 ± 0.255 ^a^
	PBB4	1.444 ± 0.126 ^c^	2.983 ± 0.191 ^b^	6.984 ± 0.299 ^b^
CCD 841 CoN	PBB1	4.708 ± 0.573 ^ab^	6.499 ± 0.198 ^b^	9.062 ± 1.195 ^a^
	PBB2	3.978 ± 0.406 ^b^	5.109 ± 0.477 ^c^	6.716 ± 0.814 ^b^
	PBB3	4.611 ± 0.452 ^ab^	6.212 ± 0.205 ^b^	9.001 ± 1.009 ^a^
	PBB4	4.989 ± 0.218 ^a^	6.921 ± 0.302 ^a^	9.594 ± 1.221 ^a^

Values marked with the same lowercase letter in the row of specific cell lines do not differ significantly *p* > 0.05.

**Table 3:** Changes in gut microbiota at different stages of the digestion process (log cfu/mL).

**Microorganisms**	**PBB1**			**PBB2**		
	**pH 7.4 ^1^**	**2 h pH 7.4 ^2^**	**18 h pH 8.0 ^3^**	**pH 7.4 ^1^**	**2 h pH 7.4 ^2^**	**18 h pH 8.0 ^3^**
*Lactobacillus*	6.461 ± 0.042	7.732 ± 0.011	9.806 ± 0.010	7.633 ± 0.014	8.756 ± 0.011	11.396 ± 0.022
*E. coli*	6.421 ± 0.058	8.234 ± 0.125	6.385 ± 0.150	7.636 ± 0.064	7.190 ± 0.020	10.046 ± 0.077
*Enterococcus*	6.622 ± 0.044	7.702 ± 0.043	6.203 ± 0.038	7.707 ± 0.090	8.611 ± 0.112	6.216 ± 0.056
*Bifidobacterium*	7.468 ± 0.125	8.369 ± 0.066	9.912 ± 0.053	7.906 ± 0.019	8.585 ± 0.024	11.257 ± 0.037
**Microorganisms**	**PBB3**			**PBB4**		
	**pH 7.4 ^1^**	**2 h pH 7.4 ^2^**	**18 h pH 8.0 ^3^**	**pH 7.4 ^1^**	**2 h pH 7.4 ^2^**	**18 h pH 8.0 ^3^**
*Lactobacillus*	7.382 ± 0.114	9.100 ± 0.056	8.686 ± 0.019	7.748 ± 0.022	8.185 ± 0.100	10.888 ± 0.044
*E. coli*	7.227 ± 0.073	6.319 ± 0.202	6.299 ± 0.062	7.736 ± 0.028	6.496 ± 0.069	9.848 ± 0.022
*Enterococcus*	7.374 ± 0.103	9.015 ± 0.065	8.677 ± 0.090	7.841 ± 0.049	7.991 ± 0.125	10.938 ± 0.042
*Bifidobacterium*	7.496 ± 0.069	8.506 ± 0.686	8.612 ± 0.030	8.011 ± 0.081	8.021 ± 0.029	10.884 ± 0.012

^1^ “in small intestine” with fecal flora; ^2^ “after small intestine”; ^3^ “after large intestine”.

**Table 4:** The results of *β*-glucuronidase and *β*-glucosidase activities after digestion (U/g of digested content).

Samples	Stage	*β*-Glucuronidase Activity	*β*-Glucosidase Activity
PBB1	Post-inoculation pH 7.4	23.96 ± 0.13 ^cb^	18.96 ± 0.33 ^b^
	2 h small intestine	20.49 ± 0.64 ^c^	19.32 ± 0.32 ^c^
	Colon onset pH 8.0	13.39 ± 0.42 ^c^	13.39 ± 0.42 ^c^
	18 h colon	18.23 ± 0.43 ^c^	18.23 ± 0.43 ^b^
PBB2	Post-inoculation pH 7.4	31.09 ± 0.36 ^b^	19.78 ± 0.19 ^b^
	2 h small intestine	25.75 ± 1.46 ^b^	25.63 ± 0.51 ^b^
	Colon onset pH 8.0	22.54 ± 0.15 ^b^	19.52 ± 0.93 ^b^
	18 h colon	11.88 ± 0.38 ^d^	15.45 ± 0.27 ^c^
PBB3	Post-inoculation pH 7.4	33.05 ± 1.59 ^a^	13.54 ± 0.17 ^c^
	2 h small intestine	21.30 ± 0.91 ^c^	17.07 ± 0.17 ^d^
	Colon onset pH 8.0	10.95 ± 0.22 ^d^	12.78 ± 0.09 ^c^
	18 h colon	28.67 ± 0.88 ^b^	31.11 ± 0.23 ^a^
PBB4	Post-inoculation pH 7.4	30.57 ± 0.92 ^b^	31.55 ± 0.64 ^a^
	2 h small intestine	46.32 ± 0.74 ^a^	42.22 ± 1.88 ^a^
	Colon onset pH 8.0	40.39 ± 0.21 ^a^	44.98 ± 1.08 ^a^
	18 h colon	33.40 ± 1.71 ^a^	31.20 ± 0.68 ^a^

Values marked with the same lowercase letter of specific samples and digestion stage do not differ significantly *p* > 0.05.

**Table 5:** Microbiological stability of plant-based burger variants (PBB1-PBB4) during 22-day refrigerated storage was assessed by monitoring the populations of mesophilic aerobic bacteria, yeasts and molds, Enterobacteriaceae, *Staphylococcus aureus*, *Clostridium perfringens*, and *Listeria monocytogenes* (log CFU/g).

Samples	Days	Total Aerobic Mesophilic Count	Yeasts & Molds	Enterobacteriaceae	Coagulase-Positive *Staphylococcus*	*C. perfringens*	*L. monocytogenes*	*L. monocytogenes* Presence	*Salmonella* Presence
PBB1	1	2.37	0.00	<0.100	<0.100	<0.100	<0.100	ND	ND
	15	2.66	0.00	<0.100	<0.100	<0.100	<0.100	ND	ND
	22	2.66	0.00	<0.100	<0.100	<0.100	<0.100	ND	ND
PBB2	1	2.24	0.00	<0.100	<0.100	<0.100	<0.100	ND	ND
	15	2.85	0.00	<0.100	<0.100	<0.100	<0.100	ND	ND
	22	2.78	0.00	<0.100	<0.100	<0.100	<0.100	ND	ND
PBB3	1	2.52	0.00	<0.100	<0.100	<0.100	<0.100	ND	ND
	15	2.88	0.00	<0.100	<0.100	<0.100	<0.100	ND	ND
	22	2.92	0.00	<0.100	<0.100	<0.100	<0.100	ND	ND
PBB4	1	2.75	0.00	<0.100	<0.100	<0.100	<0.100	ND	ND
	15	2.94	0.00	<0.100	<0.100	<0.100	<0.100	ND	ND
	22	2.86	0.00	<0.100	<0.100	<0.100	<0.100	ND	ND

ND—not detected.

## Data Availability

All data generated or analyzed during this study are included in this published article and/or Supplementary Materials.

## Supplementary Materials

The following supporting information can be downloaded at: https://www.mdpi.com/article/10.3390/nu18010160/s1, Table S1: Recipe compositions of the analyzed PBBs.
