# Supplementary material for: Potato Protein-Based Vegan Burgers: Discovering the Health-Promoting Benefits and Impact on the Intestinal Microbiome
Source: Nutrients. 2026 Jan 3;18(1):160. doi: 10.3390/nu18010160 (PMC12787374; doi:10.3390/nu18010160)
Supplement: Supplementary file 1 [file nutrients-18-00160-s001.zip › nutrients-4030596-supplementary.pdf]

Table S1. Recipe compositions of the analyzed PBBs.

Ingredient [%]	PBB1	PBB2	PBB3	PBB4
Protein base	40	40	40	40
Coconut oil	5	5	5	5
Oil blend	6	6	6	6
Potato starch	4	4	4	4
Corn starch	2	2	2	2
Yeast flakes with vitamin B_{12}	4	4	4	4
Oat flakes	4	4	4	4
Methylcellulose	2	2	2	2
Carrageenan	2	2	2	2
Aroma	2	2	2	2
Dried beetroot juice	0.75	0.75	0.75	0.75
Salt	0.5	0.5	0.5	0.5
Vinegar	0.5	0.5	0.5	0.5
Potato fiber	2	0	2	0
Oat fiber	0	2	0	2
Powdered sprouts containing ferritin	1.5	1.5	0	0
Iron (II) sulfate	0	0	0.007	0.007
Water	23.75	23.75	25.243	25.243
